# 1-(4-Fluoro­phen­yl)biguanid-1-ium chloride

**DOI:** 10.1107/S1600536810037785

**Published:** 2010-09-30

**Authors:** Maya Tutughamiarso, Michael Bolte

**Affiliations:** a Institut für Organische Chemie und Chemische Biologie, Goethe-Universität Frankfurt, Max-von-Laue-Str. 7, 60438 Frankfurt/Main, Germany; b Institut für Anorganische Chemie, Goethe-Universität Frankfurt, Max-von-Laue-Str. 7, 60438 Frankfurt/Main, Germany

## Abstract

The title compound, C_8_H_11_FN_5_^+^·Cl^−^, crystallized with a monoprotonated 1-(4-fluoro­phen­yl)biguanidinium cation and a chloride anion in the asymmetric unit. The biguanidium group is not planar [dihedral angle between the two CN_3_ groups = 52.0 (1)°] and is rotated with respect to the phenyl group [*τ* = 54.3 (3)°]. In the crystal, N—H⋯N hydrogen-bonded centrosymmetric dimers are connected into ribbons, which are further stabilized by N—H⋯Cl interactions, forming a three-dimensional hydrogen-bonded network.

## Related literature

For related structures, see: Dalpiaz *et al.* (1996 ▶); Portalone *et al.* (2004 ▶); LeBel *et al.* (2005 ▶). For hydrogen-bond motifs, see: Bernstein *et al.* (1995 ▶).
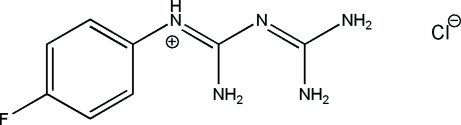


## Experimental

### 

#### Crystal data

C_8_H_11_FN_5_^+^·Cl^−^
*M* *_r_* = 231.67
Monoclinic, 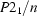

*a* = 6.9954 (5) Å
*b* = 9.2187 (4) Å
*c* = 16.3149 (11) Å
β = 91.111 (5)°
*V* = 1051.93 (11) Å^3^
*Z* = 4
Mo *K*α radiation
μ = 0.35 mm^−1^
*T* = 173 K
0.40 × 0.40 × 0.20 mm

#### Data collection

STOE IPDS II two-circle-diffractometer
13661 measured reflections
1966 independent reflections
1605 reflections with *I* > 2σ(*I*)
*R*_int_ = 0.135

#### Refinement

*R*[*F* ^2^ > 2σ(*F* ^2^)] = 0.039
*wR*(*F* ^2^) = 0.095
*S* = 1.03
1966 reflections
165 parameters
H atoms treated by a mixture of independent and constrained refinement
Δρ_max_ = 0.22 e Å^−3^
Δρ_min_ = −0.24 e Å^−3^

### 

Data collection: *X-AREA* (Stoe & Cie, 2001 ▶); cell refinement: *X-AREA*; data reduction: *X-AREA*; program(s) used to solve structure: *SHELXS97* (Sheldrick, 2008 ▶); program(s) used to refine structure: *SHELXL97* (Sheldrick, 2008 ▶); molecular graphics: *Mercury* (Macrae *et al.*, 2008 ▶)and *XP* (Sheldrick, 2008 ▶); software used to prepare material for publication: *publCIF* (Westrip, 2010 ▶).

## Supplementary Material

Crystal structure: contains datablocks I, global. DOI: 10.1107/S1600536810037785/bx2308sup1.cif

Structure factors: contains datablocks I. DOI: 10.1107/S1600536810037785/bx2308Isup2.hkl

Additional supplementary materials:  crystallographic information; 3D view; checkCIF report

## Figures and Tables

**Table 1:** Selected bond lengths (Å)

N8—C5	1.429 (2)
N8—C9	1.356 (2)
N9—C9	1.326 (2)
N10—C9	1.332 (2)
N10—C11	1.339 (2)
N12—C11	1.338 (3)
N13—C11	1.325 (3)

**Table 2:** Hydrogen-bond geometry (Å, °)

*D*—H⋯*A*	*D*—H	H⋯*A*	*D*⋯*A*	*D*—H⋯*A*
N8—H8⋯Cl^i^	0.83 (2)	2.50 (2)	3.2758 (17)	156 (2)
N9—H91⋯Cl^ii^	0.88 (3)	2.47 (3)	3.3283 (18)	163 (2)
N9—H92⋯Cl^i^	0.83 (3)	2.46 (3)	3.2358 (19)	155 (3)
N12—H121⋯Cl	0.87 (3)	2.50 (3)	3.3212 (19)	157 (2)
N12—H122⋯Cl^iii^	0.88 (3)	2.80 (3)	3.5463 (19)	143 (2)
N13—H131⋯Cl	0.87 (3)	2.58 (3)	3.384 (2)	154 (2)
N13—H132⋯N10^iv^	0.87 (3)	2.35 (3)	3.177 (3)	160 (2)

Symmetry codes: (i) 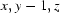; (ii) 
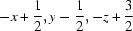
; (iii) 
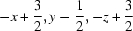
; (iv) 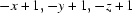.

**Fig. 1.**
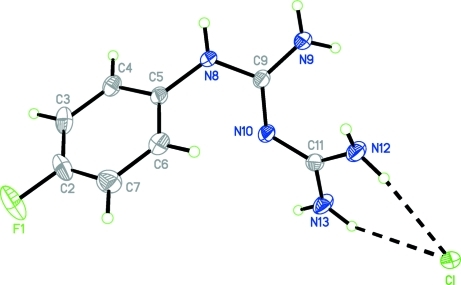
A perspective view of the title compound, showing the atom-numbering scheme. Displacement ellipsoids are drawn at the 50% probability level and H atoms are shown as small spheres of arbitrary radii. The dashed line indicates the N—H···Cl- interactions.

**Fig. 2.**
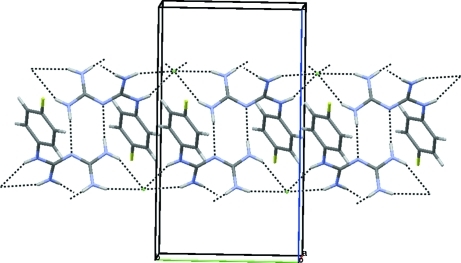
A partial packing diagram of the title compound. Hydrogen bonds are shown as dashed lines.

## supplementary crystallographic information

### Comment

1-(4-Fluorophenyl)biguanid hydrochloride crystallized with a monoprotonated 1-(4-fluorophenyl)biguanidinium cation and a chloride anion in the asymmetric unit. The protonation occurs at the N atom (N8) attached to the phenyl ring (Fig. 1). The biguanidium group is rotated with respect to the phenyl group by the rotation angle *τ* = 54.3 (3)° [the angle *τ* is defined as: *τ* = |*ω*1 + *ω*2 ± *π*|/2, the torsion angles *ω*1 and *ω*2 being respectively C4—C5—N8—C9 and C6—C5—N8—C9 (Dalpiaz *et al.*, 1996)]. The planes defined by N8, C9, N9, N10 atoms and by N10, C11, N12, N13 atoms enclose a dihedral angle of 52.0 (1)°. Similar C—N bond lengths lead to the conclusion that the *π*- electron density is delocalized over the biguanidium group (Tab. 1). Two N—H···N hydrogen bonds stabilize a centrosymmetric dimer, which is further connected to a ribbon by *R*^1^_2_(3) N—H···Cl^-^ interactions (Bernstein *et al.*, 1995; Fig. 2). The sixfold coordinated Cl^-^ anion forms another two N—H···Cl^-^ interactions leading to a three-dimensional hydrogen-bonded network.

### Experimental

Single crystals of title compound were obtained by cocrystallization of the commercially available 1-(4-fluorophenyl)biguanid hydrochloride (2.6 mg) and propylthiouracil (1.1 mg) from methanol (50 µ*L*) at room temperature.

### Refinement

All H atoms were initially located by a difference Fourier synthesis. Subsequently, H atoms bonded to aromatic C atoms were refined using a riding model, with C—H = 0.95 Å, and with *U*_iso_(H) = 1.2 *U*_eq_(C). H atoms bonded to N atoms were freely refined.

### Crystal data

**Table:**

C_8_H_11_FN_5_^+^·Cl^−^	*F*(000) = 480
*M**_r_* = 231.67	*D*_x_ = 1.463 Mg m^−^^3^
Monoclinic, *P*2_1_/*n*	Mo *K*α radiation, λ = 0.71073 Å
Hall symbol: -P 2yn	Cell parameters from 8763 reflections
*a* = 6.9954 (5) Å	θ = 3.3–26.0°
*b* = 9.2187 (4) Å	µ = 0.35 mm^−^^1^
*c* = 16.3149 (11) Å	*T* = 173 K
β = 91.111 (5)°	Block, colourless
*V* = 1051.93 (11) Å^3^	0.40 × 0.40 × 0.20 mm
*Z* = 4	

### Data collection

**Table:**

STOE IPDS II two-circle-diffractometer	1605 reflections with *I* > 2σ(*I*)
Radiation source: fine-focus sealed tube	*R*_int_ = 0.135
graphite	θ_max_ = 25.6°, θ_min_ = 3.3°
ω scans	*h* = −8→8
13661 measured reflections	*k* = −11→11
1966 independent reflections	*l* = −19→19

### Refinement

**Table:**

Refinement on *F*^2^	Secondary atom site location: difference Fourier map
Least-squares matrix: full	Hydrogen site location: inferred from neighbouring sites
*R*[*F*^2^ > 2σ(*F*^2^)] = 0.039	H atoms treated by a mixture of independent and constrained refinement
*wR*(*F*^2^) = 0.095	*w* = 1/[σ^2^(*F*_o_^2^) + (0.0521*P*)^2^] where *P* = (*F*_o_^2^ + 2*F*_c_^2^)/3
*S* = 1.03	(Δ/σ)_max_ < 0.001
1966 reflections	Δρ_max_ = 0.22 e Å^−^^3^
165 parameters	Δρ_min_ = −0.23 e Å^−^^3^
0 restraints	Extinction correction: *SHELXL*, Fc^*^=kFc[1+0.001xFc^2^λ^3^/sin(2θ)]^-1/4^
Primary atom site location: structure-invariant direct methods	Extinction coefficient: 0.008 (2)

### Special details

**Table:**

Geometry. All e.s.d.'s (except the e.s.d. in the dihedral angle between two l.s. planes) are estimated using the full covariance matrix. The cell e.s.d.'s are taken into account individually in the estimation of e.s.d.'s in distances, angles and torsion angles; correlations between e.s.d.'s in cell parameters are only used when they are defined by crystal symmetry. An approximate (isotropic) treatment of cell e.s.d.'s is used for estimating e.s.d.'s involving l.s. planes.
Refinement. Refinement of *F*^2^ against ALL reflections. The weighted *R*-factor *wR* and goodness of fit *S* are based on *F*^2^, conventional *R*-factors *R* are based on *F*, with *F* set to zero for negative *F*^2^. The threshold expression of *F*^2^ > σ(*F*^2^) is used only for calculating *R*-factors(gt) *etc*. and is not relevant to the choice of reflections for refinement. *R*-factors based on *F*^2^ are statistically about twice as large as those based on *F*, and *R*- factors based on ALL data will be even larger.

### Fractional atomic coordinates and isotropic or equivalent isotropic displacement parameters (Å^2^)

**Table:**

	*x*	*y*	*z*	*U*_iso_*/*U*_eq_	
F1	1.1564 (2)	0.20564 (19)	0.36588 (9)	0.0508 (4)	
C2	1.0273 (3)	0.1954 (3)	0.42677 (13)	0.0300 (5)	
C3	0.8876 (3)	0.0914 (2)	0.41942 (13)	0.0269 (4)	
H3	0.8799	0.0298	0.3728	0.032*	
C4	0.7580 (3)	0.0793 (2)	0.48238 (12)	0.0217 (4)	
H4	0.6598	0.0083	0.4791	0.026*	
C5	0.7707 (3)	0.16967 (19)	0.54979 (11)	0.0186 (4)	
C6	0.9161 (3)	0.2732 (2)	0.55575 (13)	0.0240 (4)	
H6	0.9266	0.3338	0.6027	0.029*	
C7	1.0446 (3)	0.2868 (2)	0.49308 (15)	0.0307 (5)	
H7	1.1430	0.3578	0.4957	0.037*	
N8	0.6343 (2)	0.15622 (17)	0.61347 (10)	0.0210 (4)	
H8	0.611 (3)	0.075 (3)	0.6328 (15)	0.021 (6)*	
C9	0.5280 (3)	0.26846 (19)	0.64102 (12)	0.0184 (4)	
N9	0.4158 (3)	0.2418 (2)	0.70383 (11)	0.0259 (4)	
H91	0.325 (4)	0.301 (3)	0.7203 (17)	0.035 (7)*	
H92	0.411 (4)	0.159 (4)	0.7249 (19)	0.043 (8)*	
N10	0.5351 (2)	0.39359 (16)	0.60039 (10)	0.0212 (4)	
C11	0.5124 (2)	0.5212 (2)	0.63811 (12)	0.0197 (4)	
N12	0.5563 (3)	0.5444 (2)	0.71722 (11)	0.0243 (4)	
H121	0.562 (4)	0.634 (3)	0.7336 (16)	0.035 (7)*	
H122	0.632 (4)	0.480 (3)	0.7417 (17)	0.037 (7)*	
N13	0.4489 (3)	0.63199 (19)	0.59356 (12)	0.0259 (4)	
H131	0.448 (4)	0.718 (3)	0.6155 (17)	0.035 (7)*	
H132	0.423 (4)	0.618 (3)	0.5418 (19)	0.036 (7)*	
Cl	0.48546 (6)	0.89909 (5)	0.73525 (3)	0.02180 (17)	

### Atomic displacement parameters (Å^2^)

**Table:**

	*U*^11^	*U*^22^	*U*^33^	*U*^12^	*U*^13^	*U*^23^
F1	0.0368 (8)	0.0794 (11)	0.0369 (8)	−0.0062 (7)	0.0206 (6)	−0.0049 (7)
C2	0.0218 (10)	0.0440 (12)	0.0245 (11)	0.0048 (9)	0.0080 (8)	0.0022 (9)
C3	0.0235 (9)	0.0357 (11)	0.0214 (10)	0.0108 (9)	−0.0021 (7)	−0.0060 (9)
C4	0.0184 (8)	0.0200 (9)	0.0265 (10)	0.0043 (7)	−0.0029 (7)	−0.0025 (8)
C5	0.0211 (9)	0.0160 (8)	0.0186 (9)	0.0053 (7)	0.0014 (7)	0.0024 (7)
C6	0.0251 (10)	0.0210 (9)	0.0259 (10)	0.0010 (8)	−0.0004 (8)	−0.0029 (8)
C7	0.0255 (10)	0.0304 (11)	0.0362 (12)	−0.0037 (9)	0.0028 (9)	0.0013 (9)
N8	0.0296 (9)	0.0119 (7)	0.0218 (8)	0.0036 (6)	0.0079 (7)	0.0022 (7)
C9	0.0213 (9)	0.0155 (9)	0.0183 (9)	0.0025 (7)	−0.0002 (7)	−0.0011 (7)
N9	0.0322 (10)	0.0164 (8)	0.0295 (10)	0.0056 (7)	0.0122 (8)	0.0029 (7)
N10	0.0305 (8)	0.0148 (7)	0.0182 (8)	0.0048 (6)	0.0030 (6)	0.0008 (6)
C11	0.0180 (9)	0.0171 (9)	0.0243 (10)	0.0019 (7)	0.0045 (7)	0.0020 (7)
N12	0.0361 (10)	0.0155 (8)	0.0211 (9)	0.0034 (8)	−0.0017 (7)	−0.0006 (7)
N13	0.0392 (10)	0.0150 (8)	0.0234 (10)	0.0055 (7)	−0.0048 (7)	0.0006 (7)
Cl	0.0265 (3)	0.0155 (2)	0.0235 (3)	−0.00112 (17)	0.00391 (17)	0.00160 (17)

### Geometric parameters (Å, °)

**Table:**

F1—C2	1.359 (2)	N8—H8	0.83 (2)
C2—C3	1.373 (3)	N9—C9	1.326 (2)
C2—C7	1.375 (3)	N10—C9	1.332 (2)
C3—C4	1.388 (3)	N9—H91	0.88 (3)
C3—H3	0.9500	N9—H92	0.83 (3)
C4—C5	1.381 (3)	N10—C11	1.339 (2)
C4—H4	0.9500	N12—C11	1.338 (3)
C5—C6	1.397 (3)	N13—C11	1.325 (3)
N8—C5	1.429 (2)	N12—H121	0.87 (3)
C6—C7	1.380 (3)	N12—H122	0.88 (3)
C6—H6	0.9500	N13—H131	0.87 (3)
C7—H7	0.9500	N13—H132	0.87 (3)
N8—C9	1.356 (2)		
			
F1—C2—C3	117.8 (2)	C9—N8—H8	116.9 (15)
F1—C2—C7	118.9 (2)	C5—N8—H8	119.4 (15)
C3—C2—C7	123.26 (19)	N9—C9—N10	125.00 (17)
C2—C3—C4	117.75 (19)	N9—C9—N8	116.83 (17)
C2—C3—H3	121.1	N10—C9—N8	118.04 (17)
C4—C3—H3	121.1	C9—N9—H91	124.2 (17)
C5—C4—C3	120.55 (18)	C9—N9—H92	121 (2)
C5—C4—H4	119.7	H91—N9—H92	113 (3)
C3—C4—H4	119.7	C9—N10—C11	121.78 (16)
C4—C5—C6	120.18 (17)	N13—C11—N12	118.33 (18)
C4—C5—N8	119.54 (17)	N13—C11—N10	117.81 (19)
C6—C5—N8	120.28 (17)	N12—C11—N10	123.82 (17)
C7—C6—C5	119.64 (19)	C11—N12—H121	117.2 (18)
C7—C6—H6	120.2	C11—N12—H122	117.0 (17)
C5—C6—H6	120.2	H121—N12—H122	118 (3)
C2—C7—C6	118.6 (2)	C11—N13—H131	119.0 (19)
C2—C7—H7	120.7	C11—N13—H132	118.7 (17)
C6—C7—H7	120.7	H131—N13—H132	122 (3)
C9—N8—C5	123.55 (16)		
			
F1—C2—C3—C4	178.81 (18)	C5—C6—C7—C2	−1.1 (3)
C7—C2—C3—C4	0.2 (3)	C4—C5—N8—C9	−125.8 (2)
C2—C3—C4—C5	0.1 (3)	C6—C5—N8—C9	54.3 (3)
C3—C4—C5—C6	−0.9 (3)	C5—N8—C9—N9	−176.00 (18)
C3—C4—C5—N8	179.33 (16)	C5—N8—C9—N10	8.0 (3)
C4—C5—C6—C7	1.4 (3)	N9—C9—N10—C11	34.9 (3)
N8—C5—C6—C7	−178.78 (18)	N8—C9—N10—C11	−149.46 (18)
F1—C2—C7—C6	−178.25 (19)	C9—N10—C11—N13	−154.64 (18)
C3—C2—C7—C6	0.3 (3)	C9—N10—C11—N12	27.7 (3)

### Hydrogen-bond geometry (Å, °)

**Table:**

*D*—H···*A*	*D*—H	H···*A*	*D*···*A*	*D*—H···*A*
N8—H8···Cl^i^	0.83 (2)	2.50 (2)	3.2758 (17)	156 (2)
N9—H91···Cl^ii^	0.88 (3)	2.47 (3)	3.3283 (18)	163 (2)
N9—H92···Cl^i^	0.83 (3)	2.46 (3)	3.2358 (19)	155 (3)
N12—H121···Cl	0.87 (3)	2.50 (3)	3.3212 (19)	157 (2)
N12—H122···Cl^iii^	0.88 (3)	2.80 (3)	3.5463 (19)	143 (2)
N13—H131···Cl	0.87 (3)	2.58 (3)	3.384 (2)	154 (2)
N13—H132···N10^iv^	0.87 (3)	2.35 (3)	3.177 (3)	160 (2)

Symmetry codes: (i) *x*, *y*−1, *z*; (ii) −*x*+1/2, *y*−1/2, −*z*+3/2; (iii) −*x*+3/2, *y*−1/2, −*z*+3/2; (iv) −*x*+1, −*y*+1, −*z*+1.
